# *Urtica dioica* From El Menzel (Morocco): Phytochemical Analysis, In Vivo and In Silico Evaluation of Analgesic and Anti‐Inflammatory Effects, and Toxicological Study With ADME Profiling

**DOI:** 10.1002/fsn3.71253

**Published:** 2025-11-30

**Authors:** Sara Tlemcani, Amal Lahkimi, Oussama Khibech, Amal Elrherabi, Mohamed Bouhrim, Fahd A. Nasr, Mohammed Al‐zharani, Ashraf Ahmed Qurtam, Meryem Doubi, Hicham Bekkari

**Affiliations:** ^1^ Laboratory of Biotechnology, Environment, Agri‐Food, and Health, Faculty of Sciences Dhar El Mahraz Sidi Mohamed Ben Abdellah University Atlas Morocco; ^2^ Laboratory of Engineering, Molecular Organometallic Materials and Environment, Faculty of Sciences Dhar El Mahraz Sidi Mohamed Ben Abdellah University Atlas Morocco; ^3^ Laboratory of Applied Chemistry and Environment (LCAE), Faculty of Science University Mohammed Premier Oujda Morocco; ^4^ Laboratory of Bioresources, Biotechnology, Ethnopharmacology and Health, Faculty of Sciences University Mohammed 1st Oujda Morocco; ^5^ Biological Engineering Laboratory, Faculty of Sciences and Techniques Sultan Moulay Slimane University Beni Mellal Morocco; ^6^ Biology Department, College of Science Imam Mohammad Ibn Saud Islamic University (IMSIU) Riyadh Saudi Arabia; ^7^ Laboratory of Natural Resources and Sustainable Development, Faculty of Sciences Ibn Tofail University – KENITRA – University Campus Kenitra Morocco

**Keywords:** analgesic activity, anti‐inflammatory, HPLC, in silico, in vitro, polyphenolic extract, toxicity, Utrica dioica

## Abstract

Stinging nettle ( *Urtica dioica* ) remains under‐characterized despite its widespread ethnomedicinal use. Here, we combine quantitative phytochemistry, validated rodent pharmacology, and multi‐target molecular modeling to explore the analgesic and anti‐inflammatory potential of a Moroccan ecotype. HPLC‐PDA analysis of the hydro‐ethanolic extract (yield 14.8%) identified 15 phenolics, dominated by chlorogenic acid (26.5%), caffeic acid (18.8%), and p‐coumaric acid (12.8%). In vivo, the polyphenolic fraction (100 mg/kg, p.o.) significantly reduced acetic‐acid writhing and carrageenan paw edema by 52% and 73%, respectively, and decreased both neurogenic and inflammatory phases of the formalin assay by over 59%. Similar efficacy was observed with the crude extract at 400 mg/kg, and acute oral toxicity tests showed no adverse effects up to 4 g/kg. Docking studies to mPGES‐1, TRPV1, and BK2R revealed binding energies as low as −10.5 kcal/mol, highlighting rutin, hyperoside, and chlorogenic acid as promising lead compounds. ADME/Tox profiling indicated high oral developability for apigenin and a broad safety margin for all leads. Overall, these findings position *U. dioica* extracts as mechanistically validated phytopharmaceutical candidates for pain and inflammation management, supporting their potential for clinical development.

## Graphical abstract

The hydro‐ethanolic extract of *Urtica dioica* (yield 14.8%) contained 15 phenolics, mainly chlorogenic, caffeic, and p‐coumaric acids. The polyphenolic fraction (100 mg/kg, p.o.) significantly reduced acetic‐acid writhing (52%) and carrageenan‐induced edema (73%) without toxicity up to 4 g/kg. Docking studies showed strong affinities of rutin, hyperoside, and chlorogenic acid for mPGES‐1, TRPV1, and BK2R, supporting *U. dioica* as a safe, mechanistically validated candidate for pain and inflammation management.

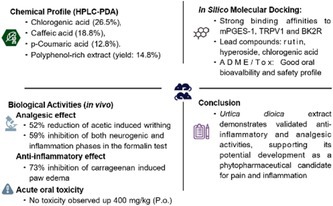

## 1 Introduction

*Urtica dioica* , commonly known as stinging nettle, belongs to the family *Urticaceae* and is widely distributed in temperate regions, including extensive growth in Morocco. The plant develops phloem fibers rich in active constituents and crystalline cellulose (Bacci et al. 2009; Farag et al. 2013). Contact with *U. dioica* causes a burning sensation due to the injection of histamine and other chemicals through specialized hair‐like structures called trichomes, which are located on the stems and leaves and function similarly to hypodermic needles (Majedi et al. 2021). In Morocco, *U. dioica* is widely used as a botanical remedy in traditional medicine. The aerial parts of the plant, including leaves, stems, and seeds, are commonly used for extraction due to their high content of phenolic compounds, which exhibit potent antioxidant properties (Shan et al. 2009). Traditionally, the plant is used to treat dermatological and digestive disorders (Tlemcani et al. 2023), as well as rheumatic pain, colds, and coughs (Kregiel et al. 2018). Furthermore, it is employed in the treatment of arthritis, lumbago, and paralysis, and is believed to enhance blood circulation (Upton 2013). Studies have also shown that *U. dioica* may contribute to the protection and regeneration of liver tissue and help prevent hepatic damage (Oguz et al. 2015). *U. dioica* contains a variety of chemical constituents, including proteins, amino acids, saponins, and flavonoids. The concentration of these compounds is influenced by several factors, such as edaphic conditions, the timing of harvest, extraction methods, and the solvents used (Đurović et al. 2017). Recent studies have also explored the potential of such compounds in developing new antimicrobial agents for the treatment of nosocomial infections (Gülçin et al. 2004; Shah 2005). Pain is a multifaceted physiological response involving both peripheral and central mechanisms, and its effective management remains a major challenge in clinical settings. Analgesic agents are evaluated through a variety of experimental models to determine their efficacy and mechanisms of action (Le Bars et al. 2001). Among these, the acetic acid‐induced writhing and formalin tests are widely used for assessing peripheral and central nociceptive responses, respectively. The acetic acid test reflects inflammatory pain through peripheral mechanisms involving prostaglandin pathways, while the formalin test includes an early neurogenic phase followed by a later inflammatory phase, allowing differentiation of analgesic drug types. These methods have become indispensable in the preclinical screening of natural products with putative analgesic effects (Hunskaar and Hole 1987). Inflammation is a complex biological response that underlies many acute and chronic diseases. To evaluate potential anti‐inflammatory agents, experimental models such as carrageenan‐induced paw edema are commonly employed. The carrageenan (CA)‐induced paw edema model, first established by Winter et al. (1962) remains one of the most widely used experimental methods for assessing the anti‐inflammatory potential of new substances, having played a pivotal role in the discovery of nonsteroidal anti‐inflammatory drugs (NSAIDs) and selective COX‐2 inhibitors. This model induces a localized acute inflammatory response characterized by classic signs such as edema, pain sensitivity (hyperalgesia), and redness (Morris 2003). The inflammatory process occurs in two distinct phases: an initial phase involving the release of mediators such as histamine, serotonin, and bradykinin, followed by a delayed phase dominated by leukocyte infiltration, prostaglandin production, and inducible cyclooxygenase (COX‐2) activity (Guay et al. 2004). Cytokines play an essential role in this response. These small signaling proteins regulate cellular communication and immune responses, and are deeply involved in the pathogenesis of several chronic inflammatory diseases such as asthma, multiple sclerosis, rheumatoid arthritis, and inflammatory bowel disease (McInnes and Schett 2007). Pro‐inflammatory cytokines such as tumor necrosis factor‐alpha (TNF‐α), interleukin‐1β (IL‐1β), and interleukin‐6 (IL‐6) are particularly implicated in edema formation, leukocyte recruitment, and the development of pain hypersensitivity in this model (Rocha et al. 2006). Despite progress in developing cytokine‐targeting therapies such as Infliximab and Tocilizumab (Smolen and Aletaha 2011), current treatments often carry significant side effects and costs. As such, research continues to explore natural bioactive compounds capable of modulating cytokine activity as safer alternatives (Vazquez et al. 2015). The originality of the present study lies in the exploration of *U. dioica* from a Moroccan region (El Menzel) where the plant remains underexploited both scientifically and therapeutically, despite its widespread traditional use. In addition, while various extraction methods have been applied to this species in other contexts, the use of a hydroethanolic extract remains largely unexplored for Moroccan populations. This approach is particularly relevant, as hydroethanolic solvents are known to enhance the extraction of a broader range of polar and semi‐polar bioactive compounds, potentially increasing the pharmacological value of the extract. Moreover, the integrative design of this study, combining in vivo pharmacological evaluation with *in silico* approaches such as ADME prediction (Touam et al. 2025), toxicity profiling, and molecular docking, offers a comprehensive investigation into the pharmacodynamic potential and safety profile of *U. dioica* bioactives. Despite its recognized traditional use, there remains a lack of comprehensive scientific validation of *U. dioica* 's analgesic and anti‐inflammatory potential using both biological and computational tools, particularly in the context of Moroccan ecotypes. Accordingly, the present study aims to evaluate the chemical composition, analgesic, and anti‐inflammatory effects of *Urtica* species with a focus on promoting health, preventing disease, and supporting overall well‐being.

## 2 Materials and Methods

### 2.1 Plant Samples Harvest

The plant samples were collected from El Menzel Province Sefrou (Latitude: 33 50′ 11.99″ N Longitude: −4 32′ 26.99″ W), located in the Fes‐Meknes region of Morocco, between March and April, coinciding with their peak growth and flowering period. The species was identified and authenticated by botanist Eloutassi Nouredinne, a professor at the Regional Center for Education and Training in Fez, under the voucher specimen number FMM/032102. The identification was accredited by the French Association for Plant Protection.

### 2.2 Solvent Extraction Methods

The hydroethanolic extract was obtained through maceration. Twenty grams of plant powder, previously rinsed and dried in an oven at 45°C for 72 h, were macerated for 48 h in a solvent mixture of 70% ethanol and 30% distilled water. The resulting solution was filtered using Whatman paper, and the solvent was subsequently removed by evaporation under reduced pressure using a rotary evaporator (BUCHI R‐100 Rotavapor, New Castle, DE, USA) (Tlemcani et al. 2023). The final crude extract was stored at 6°C in Eppendorf tubes for further use. The extraction yield was 14.8%.

### 2.3 Polyphenolic Extraction

For this extraction, 10 g of *U. dioica* powder was macerated three times in 30 mL of methanol. The macerate was then filtered and concentrated at a low temperature. The concentrated extract was dissolved in 50 mL of distilled water and subjected to liquid–liquid extraction using 20 mL of hexane, chloroform, and ethyl acetate, each performed three times. The ethyl acetate phase was evaporated under vacuum at 40°C using a rotary evaporator to obtain the polyphenolic fraction. The extraction yield of the polyphenolic extract was 5.89%.

### 2.4 Phytochemical Analysis With HPLC

Analytical samples were analyzed using Waters Alliance HPLC (e2695 separation module), equipped with 2998 Photodiode Array (PDA). Data acquisition and control were carried out using Empower 3 chromatography software (Waters, Germany). The preparative high‐pressure liquid chromatography (Prep‐HPLC) system consisted of 3535 modules quaternary gradient equipped with 996 PDA detectors. HPLC was performed under reversed phase conditions using a TSQ Quantum Access MAX (Thermo Scientific), which includes a Dionex pump with a degassing module, an Accela PDA detector and an Accela autosampler. The separations chromatographies were carried out on a Kinetex column (C8, particle size 2.6 μm, pore size 100 Å, 100 × 2.1 mm). One UHPLC Security Guard cartridge (C8, for ID 2.1 mm column). The injection volume was 10 μL; the oven temperature was maintained at 35°C.

### 2.5 Animals

To evaluate the pharmacological activities, male Wistar rats weighing 100–150 g were selected for the analgesic and anti‐inflammatory tests. For the toxicity tests, Swiss albino mice weighing between 25 and 30 g were used. The animals were housed in a controlled environment with a relative humidity of 50%–55% and a temperature maintained at 22°C ± 2°C. They had free access to food and water under a 12/12‐h light/dark cycle. All experimental procedures were conducted under ethical guidelines for the care and use of laboratory animals (Institute for Laboratory Animal Research (US), Committee for the Update of the Guide for the Care and Use of Laboratory Animals 1986).

### 2.6 Acute Analgesic Activity Evaluation In Vivo

#### 2.6.1 Acetic Acid Method

This experiment was conducted according to the method described by Konaté et al. (2012), which assesses the analgesic potential of a substance by evaluating its ability to reduce pain‐induced writhing in rats following the injection of an irritant. The irritant used, acetic acid, induces abdominal constrictions, which serve as a measure of nociceptive response. A total of 30 Wistar rats were randomly assigned to six groups, each consisting of five animals (*n* = 5). Group 1 (positive control) received Paracetamol (200 mg/kg), Group 2 (negative control) received NaCl 0.9% (10 mL/kg), Groups 3 and 4 received the hydroethanolic extract at doses of 200 and 400 mg/kg, respectively, Group 5 and 6 received polyphenolic extract at 50 and 100 mg/kg, respectively. One hour after administration of the respective treatments, all rats were injected intraperitoneally with 1% acetic acid (10 mL/kg body weight). Following a latency period of 5 min, the number of abdominal constrictions was recorded for each animal over a 30‐min observation period.

#### 2.6.2 Formalin Test

The formalin test was conducted to evaluate pain response according to the method described by Coderre et al. (1993), this model assesses both neurogenic and inflammatory pain components through observation of behavioral responses following a formalin injection. Thirty Wistar rats were randomly divided into six groups, each containing five animals (*n* = 5). The animals received oral treatments with either Tramadol (used as the reference drug) or plant extracts. Thirty minutes post‐treatment, each rat was subcutaneously injected with 20 μL of 2% formalin into the plantar surface of the right hind paw. Pain‐related behavior was assessed by measuring the duration of paw licking and biting, which was recorded using a stopwatch. The response was observed in two distinct phases: The first phase (0–5 min) and the second phase (15–30 min) represent the total number of seconds spent licking the formalin‐injected paw. Thirty Wistar rats were separated into six groups with five animals per group (*n* = 5). Group 1 (Positive control) received Paracetamol 200 mg/kg, Group 2 (Negative control) received 0.9% NaCl, Group 3 and 4 received hydro‐ethanol extract solution at 200 and 400 mg/kg, respectively, Group 5 and 6 received polyphenolic extract at 50 and 100 mg/kg, respectively.

### 2.7 Evaluation of Acute Anti‐Inflammatory Activity

The anti‐inflammatory activity was evaluated using the carrageenan‐induced paw edema model in rats, following the method described by Manouze et al. (2017). Before the experiment, the animals were fasted for 12 h, weighed, and randomly assigned into six groups (*n* = 5 per group). Group 1 (Positive Control) received diclofenac at a dose of 15 mg/kg, Group 2 (Negative Control) received 0.9% sodium chloride solution (NaCl), Groups 3 and 4 received hydroethanolic extract of *U. dioica* at doses of 200 and 400 mg/kg, respectively, Groups 5 and 6 received polyphenolic extract of *U. dioica* at doses of 50 and 100 mg/kg, respectively. One hour after treatment, each rat received a subcutaneous injection of 5 μL of 1% carrageenan solution into the plantar surface of the right hind paw. Paw volumes were measured using a plethysmometer at the following time points: 1 h before carrageenan injection (*V*_0_), then 3 h (T3), 4 h (T4), 5 h (T5), 6 h (T6) and after carrageenan injection (*V*_*t*_).

The inhibition percentage of edema (EI) was calculated as stated below:

$$\begin{array}{c} \text{Inhibition}\%{\left(\left(\left(V_{t}-V_{0}\right)\;\text{Control}-\left(V_{t}-V_{0}\right)\;\text{Treated}\right)/\left(V_{t}-\text{V0}\right)\;\text{Control}\right)}^{*}\;\\ 100 \end{array}$$

*V*_*t*_: The volumes after carrageenan administration, *V*_0_: The volumes before carrageenan administration.

### 2.8 Acute Toxicity Test

The acute toxicity of the hydro‐methanolic extract of *Urtica dioica* was evaluated in mice following the procedure described by Hilan et al. (2009). The extract was administered orally at doses of 600, 1000, 2000, and 4000 mg/kg, diluted in physiological saline solution (0.9% NaCl). A total of 30 mice were randomly divided into five groups, with six animals per group (*n* = 6). Group 1 (Negative Control) received only the physiological saline solution, Groups 2 to 5 received increasing doses of the *U. dioica* extract (600, 1000, 2000, and 4000 mg/kg, respectively). Following administration, the animals were observed continuously for 72 h to monitor any signs of toxicity or mortality. Observations included behavioral changes, physical activity, and survival rate, in order to assess the safety profile of the extract.

### 2.9 Blind Molecular Docking

Crystal structures of mPGES‐1 (PDB 4YL3), TRPV1 (PDB 5IRZ) and BK2R (PDB 7F6H) were retrieved from the Protein Data Bank. All non‐essential hetero‐atoms were removed and polar hydrogens plus Kollman charges were added with AutoDock Tools 1.5.7, after which each receptor was saved as a pdbqt file. The 15 phytochemicals previously characterized by HPLC were downloaded from PubChem in sdf format, converted to three‐dimensional geometry, energy‐minimized, and exported to pdbqt via the Open Babel interface in PyRx 0.9.9. Blind docking that is, an unbiased search that covers the entire protein surface, was then executed with the AutoDock Vina module built into PyRx (Trott and Olson 2010); the top‐scoring pose for each ligand‐protein pair was retained for subsequent interaction analysis.

### 2.10 In Silico ADME Assessment

SwissADME was employed to obtain a rapid, rule‐based assessment of the developability of chlorogenic acid, apigenin and rutin (Trott and Olson 2010). Canonical SMILES for each compound were pasted into the web interface, which automatically calculates key physicochemical descriptors (lipophilicity, polarity, solubility) and applies established filters for oral drug‐likeness, gastrointestinal absorption and blood–brain penetration. The resulting summary tables and radar plots underpin the ADME discussion.

### 2.11 In Silico Acute‐Toxicity Prediction

SMILES strings were also submitted to ProTox‐III to estimate oral LD_50_ values and assign OECD toxicity classes (Banerjee et al. 2024), providing an early indication of acute safety. The platform's default consensus model was used without manual adjustment, and the predicted classes were compared across the three lead compounds.

### 2.12 Data Analysis

The results were expressed as mean ± standard deviation (SD) to represent data variability. Multiple group comparisons were performed using one‐way analysis of variance (ANOVA), which allows the determination of statistically significant differences between groups. All data were processed and analyzed using Microsoft Excel, employed as a tool for data management and statistical analysis.

## 3 Results

### 3.1 Phytochemical Compounds of *U. dioica* Hydroethanolic Extract

**FIGURE 1:**
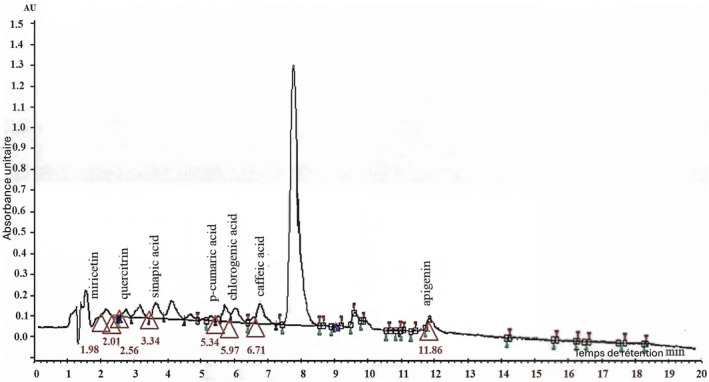
HPLC chromatogram of the hydroethanolic extract of *Urtica dioica* at 320 nm, with overlaid UV–Vis spectra recorded between 210 and 500 nm.

Figure 1 presents the chromatogram obtained through high‐performance liquid chromatography (HPLC), while Table 1 provides a precise quantification of the identified compounds, allowing for a detailed analysis of their presence and concentration in the sample. According to the HPLC analysis of the hydroethanolic extract of *Urtica dioica* , the predominant compounds were chlorogenic acid (26.48%) with a retention time of 5.97 min, caffeic acid (18.78%) with a retention time of 6.71 min, and *p*‐coumaric acid (12.75%) with a retention time of 5.34 min (Table 1). The chromatographic analysis also quantified other significant compounds, including ferulic acid (7.39%), sinapic acid (7.00%), apigenin (7.00%), and rutin (5.73%), all of which were present at noteworthy concentrations. Additionally, several flavonoids were identified in the extract, such as quercetin, kaempferol, and luteolin, further indicating the rich polyphenolic content of *U. dioica* .

**TABLE 1:** Identification and quantification of compounds in the *Urtica dioica* extract by HPLC analysis.

Compound	TR (min)	(%)
Sinapic acid	3.34	7.0091
Apigenin	11.25	7.0009
Quercitrin	2.56	2.0124
Isoquercitrin	7.54	1.2855
Rutin	10.35	5.7382
Hyperoside	2.01	1.0043
Caffeic acid	6.71	18.7893
Ferulic acid	11.23	7.3977
p‐Cumaric acid	5.34	12.7542
Patuletin	7.53	2.0238
Kaempferol	9.18	1.2711
Luteolin	8.09	1.1329
Chlorogenic acid	5.97	26.4832
Fisetin	10.23	1.3292
Gentisic acid	10.58	3.0429

In a previous study, it was revealed that the hydroethanolic extract of *Urtica dioica* leaves contains 13 phenolic compounds identified by HPLC‐DAD‐ESI/MS^2^. The most abundant compounds were rutin, followed by caffeoylquinic acid isomers, particularly compound 6. Other notable flavonoids included kaempferol‐O‐rutinoside, isorhamnetin‐O‐rutinoside, and kaempferol‐O‐hexoside. Caffeoyl tartaric acid and isorhamnetin dihexoside malonate were reported in *U. dioica* for the first time. Identified phenolics, especially caffeoylquinic acids and flavonoid glycosides, contribute to the plant's known antioxidant, anti‐inflammatory, antidiabetic, and anti‐obesity properties, aligning with previous studies highlighting the therapeutic potential of these compounds (Altamimi et al. 2022). The HPLC–DAD profiling of the crude aqueous extract of *Urtica dioica* revealed a rich phytochemical composition dominated by hydroxycinnamic acid derivatives, flavonoids, and anthocyanins. The most abundant compounds were rutin (3.209 ± 1.151 mg/g), 2‐O‐caffeoylmalic acid (2.059 ± 1.015 mg/g), chlorogenic acid (2.039 ± 0.073 mg/g), and isorhamnetin 3‐O‐rutinoside (2.675 ± 0.312 mg/g), highlighting the extract's high antioxidant potential. The detection of anthocyanins such as peonidin 3‐O‐rutinoside and rosinidin 3‐O‐rutinoside further supports the pharmacological relevance of *U. dioica* , particularly in oxidative stress‐related disorders (Dar et al. 2013).

The use of hydroethanolic maceration enhances the extraction of both water and solvent‐soluble bioactive compounds, optimizing the phytochemical yield. Phytochemical screening revealed that *Urtica dioica* extracts are rich in phenolic compounds, particularly chlorogenic acid and caffeic acid, both known for their antioxidant and anti‐inflammatory properties.

### 3.2 Acute Analgesic Activity In Vivo

#### 3.2.1 Acetic Acid Method

**FIGURE 2:**
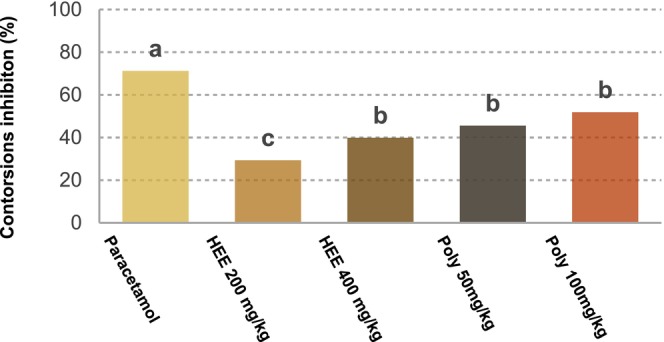
Percentage inhibition of acetic acid‐induced writhing in rats following treatment with paracetamol and *Urtica dioica* extracts (*n* = 5). (a, b, c) represent treatment differences that are statistically significant at *p* < 0.05. HEE 200 mg/kg: Hydroethanolic extract at 200 mg/kg; HEE 400 mg/kg: Hydroethanolic extract at 400 mg/kg; Poly 50 mg/kg: Polyphenolic extract at 50 mg/kg; Poly 100 mg/kg: Polyphenolic extract at 100 mg/kg.

The number of contortions under different treatments is presented in Table 2. Contortion percentage inhibition is illustrated in Figure 2. The control group showed a high number of contortions with a mean number of writhes of 70.2 ± 2.97. Paracetamol, used as a positive control at a dose of 10 mg/kg, significantly reduced the number of contortions to 20.2 ± 2.5 compared to the control group with a reduction of 71.22%. For hydroethanolic extracts (HEE), at a dose of 200 mg/kg, the inhibition rate of writhing is approximately 29.34%. At a higher dose of 400 mg/kg, the inhibition increases to approximately 39.88%. For polyphenolic extracts (Poly), at doses of 50 and 100 mg/kg, the inhibition rates of writhing are approximately 45.58% and 51.85%, respectively.

**TABLE 2:** Effect of *U. dioica* extracts on the number of writhing responses in rats following acetic acid injection (*n* = 5).

Treatments	Number of contortions
	*U. dioica*
Control	70.2 ± 2.97
Paracetamol (10 mg/kg)	20.2 ± 2.52
HEE (200 mg/kg)	49.6 ± 2.16
HEE (400 mg/kg)	42.2 ± 1.88
Poly (50 mg/kg)	38.2 ± 1.66
Poly (100 mg/kg)	33.8 ± 1.85

A previous study also revealed the analgesic potential of *Urtica dioica* using the acetic acid‐induced writhing test in albino mice. The aqueous leaf extract significantly reduced the number of writhes, especially at a dose of 150 mg/kg, indicating a notable peripheral analgesic effect (Safari et al. 2016). These findings support the traditional use of the plant for pain relief and are consistent with the analgesic effects observed in our extracts, particularly the polyphenolic fraction at 100 mg/kg, which showed a higher inhibition rate than the hydroethanolic extract. In addition, another study on *Urtica dioica* hydroalcoholic leaf extract demonstrated a dose‐dependent analgesic effect in the acetic acid‐induced writhing test in mice. The extract, administered at doses of 100, 200, and 400 mg/kg, reduced abdominal writhing by 41%, 64%, and 81%, respectively, while indomethacin, used as a reference drug, achieved 84% inhibition (Hajhashemi and Klooshani 2013).

#### 3.2.2 Formalin Test

**FIGURE 3:**
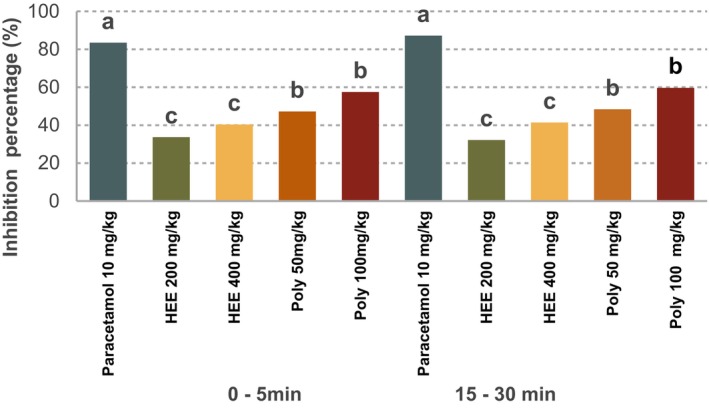
Percentage of pain inhibition in rats following formalin‐induced nociception after treatment with *Urtica dioica* extracts and paracetamol. HEE 200 mg/kg: Hydroethanolic extract at 200 mg/kg; HEE 400 mg/kg: Hydroethanolic extract at 400 mg/kg; Poly 50 mg/kg: Polyphenolic extract at 50 mg/kg; Poly 100 mg/kg: Polyphenolic extract at 100 mg/kg. Different letters (a, b, c) indicate statistically significant differences between treatment groups (*p* < 0.05).

The results of the formalin test, expressed as paw‐licking time (in seconds), demonstrate the analgesic activity of the various treatments. Table 3 summarizes the effects of each treatment group, including the negative control, standard drug (Paracetamol), hydroethanolic extracts (HEE) at doses of 200 and 400 mg/kg, and polyphenolic extracts (Poly) at doses of 50 and 100 mg/kg. The percentage inhibition of pain is presented in Table 3 and illustrated graphically in Figure 3. In the early phase (0–5 min), the hydroethanolic extract produced pain inhibition of 33.67% at 200 and 40.35% at 400 mg/kg. In the late phase (15–30 min), inhibition increased to 32.17% and 41.41% for the respective doses. The polyphenolic extract showed even stronger analgesic effects: at 50 mg/kg, it inhibited pain by 47.21% in the early phase and 48.39% in the late phase; at 100 mg/kg, the inhibition reached 57.48% in the early phase and 59.64% in the late phase. Paracetamol, used as the standard treatment, exhibited the most potent effect, achieving a maximum pain inhibition of 87.23% across both phases. In a previous investigation, the analgesic activity of *Urtica dioica* was also evaluated using the formalin‐induced pain model. The aqueous extract significantly reduced paw‐licking behavior in both the early neurogenic phase and the late inflammatory phase. The effect was dose‐dependent, with higher doses showing a stronger reduction in pain response, particularly in the second phase, highlighting its potential anti‐inflammatory and analgesic properties (Safari et al. 2016). These results align with our findings, especially the effects observed with the polyphenolic extract at 100 mg/kg, which exhibited a comparable inhibition profile in both phases of the formalin test.

**TABLE 3:** Effect of hydroethanolic and polyphenolic extracts of *U. dioica* on formalin‐induced pain in rats (*n* = 5). Values represent the mean licking time (in seconds) recorded during the early phase (0–5 min) and late phase (15–30 min) after formalin injection.

Treatments	Licking time (second)	
	Fist phase (0–5 min)	Second phase (15–30 min)
Control	56.00 ± 1.58	31.20 ± 0.83
Paracetamol (10 mg/kg)	9.49 ± 1.34	4.00 ± 0.57
HEE (200 mg/kg)	37.14 ± 0.22	21.16 ± 0.22
HEE (400 mg/kg)	33.4 ± 0.66	18.28 ± 0.83
Poly (50 mg/kg)	29.56 ± 0.15	16.10 ± 0.13
Poly (100 mg/kg)	23.81 ± 0.51	12.59 ± 0.24

The analgesic activity observed in the acetic acid and formalin tests confirms a dose‐dependent response, with polyphenolic extracts demonstrating stronger effects than hydroethanolic ones. This suggests that phenolics, especially flavonoids such as rutin, apigenin, quercetin, and kaempferol, contribute significantly to the observed pain relief. Notably, the polyphenolic extracts (50 and 100 mg/kg) were more effective than lower doses of hydroethanolic extract, statistically confirmed by the observed treatment differences (*p* < 0.05). Paulauskienė and colleagues showed that polyphenols extracts possess significant bioactive properties, supporting these observations (Paulauskienė et al. 2021). Polyphenols have demonstrated significant peripheral analgesic activity in vivo. In pain models such as the acetic acid‐induced writhing test, polyphenol‐rich fractions including flavonoids and tannins produced over 50% inhibition of nociceptive responses. These results suggest that polyphenols contribute meaningfully to pain relief, likely through modulation of inflammatory mediators involved in peripheral nociception (Garcia et al. 2015). The dual‐phase formalin test highlighted the capacity of *U. dioica* extracts to reduce both neurogenic and inflammatory pain responses. These effects are likely linked to modulation of key mediators like prostaglandins, serotonin, and histamine (Haley et al. 1989). The observed analgesic potential, although lower than paracetamol, was significant and dose‐responsive.

### 3.3 Acute Anti‐Inflammatory Activity

**FIGURE 4:**
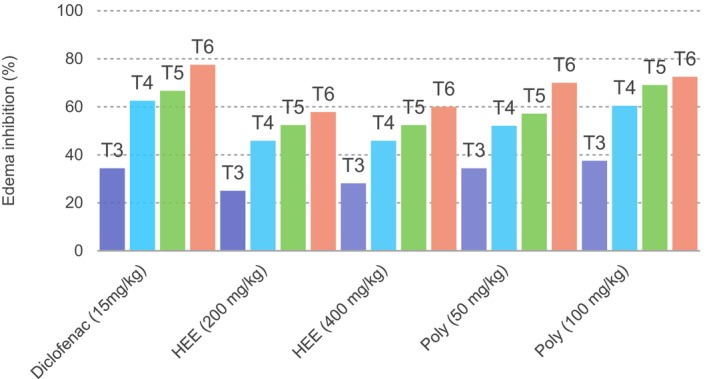
Percentage inhibition of carrageenan‐induced paw edema in rats treated with *Urtica dioica* extracts and reference drug. HEE 200 mg/kg: Hydroethanolic extract at 200 mg/kg; HEE 400 mg/kg: Hydroethanolic extract at 400 mg/kg; Poly 50 mg/kg: Polyphenolic extract at 50 mg/kg; Poly 100 mg/kg: Polyphenolic extract at 100 mg/kg. (T3): 3 h after carrageenan injection, (T4): 4 h after carrageenan injection, (T5): 5 h after carrageenan injection, (T6): 6 h after carrageenan injection.

The anti‐inflammatory activity of *Urtica dioica* extracts was assessed using the carrageenan‐induced paw edema model in rats. Hydroethanolic extracts (HEE) at 200 mg/kg and 400 mg/kg showed progressive reductions in paw diameter, starting at 2.13 cm and 2.12 cm, respectively, and reaching 2.30 cm and 2.28 cm by T6. These reductions were consistent across all intermediate time points. Similarly, polyphenolic extracts (Poly) at 50 mg/kg and 100 mg/kg demonstrated dose‐dependent anti‐inflammatory effects, with final paw diameters of 2.20 cm and 2.21 cm, respectively, at T6 (Table 4). The percentage of edema inhibition is illustrated in Figure 4. The most pronounced anti‐inflammatory effect was observed with the polyphenolic extract at 100 mg/kg, showing edema inhibition rates of 60.41%, 69.04%, and 72.50% at T4, T5, and T6, respectively. The polyphenolic extract at 50 mg/kg exhibited inhibition rates of 34.37%, 52.08%, 57.14%, and 70.00% at T3, T4, T5, and T6, respectively. Hydroethanolic extracts at 200 mg/kg and 400 mg/kg showed comparable effects, with inhibition percentages of 25.00%, 45.83%, 52.38%, and 57.80% for 200 mg/kg, and 28.12%, 45.83%, 52.38%, and 60.00% for 400 mg/kg at T3 through T6, respectively. In a study by Tekin et al. (2009), the anti‐inflammatory effects of *Urtica dioica* L. fixed oil were evaluated using the carrageenan‐induced paw edema model in rats. The results indicated that both doses of *U. dioica* fixed oil (0.05 and 0.15 mL/kg) significantly reduced inflammation by 47.4% and 57.0%, respectively, compared to the control group. However, these effects were less potent than the 95.7% reduction achieved with the standard anti‐inflammatory drug, indomethacin (3 mg/kg). Overall, the study concluded that *U. dioica* fixed oil possesses mild anti‐inflammatory properties but is not as effective as indomethacin in this model (Tekin et al. 2009). Another study showed that the anti‐inflammatory effects of *Urtica dioica* essential oil (UDEO) were evaluated using the carrageenan‐induced paw edema model in rats. The results indicated that both UDEO and indomethacin significantly reduced paw edema, with UDEO exhibiting a comparable effect to the standard anti‐inflammatory drug. Histopathological examinations of the paws revealed minimal infiltration of inflammatory cells in UDEO‐treated rats, further supporting its anti‐inflammatory properties. These effects are attributed to the high content of phenolic and flavonoid compounds in UDEO, which possess antioxidant and anti‐inflammatory activities (Tekin et al. 2009). In addition, Safari et al. (2016) investigated the anti‐inflammatory effects of a *Urtica dioica* leaf extract using the carrageenan‐induced paw edema model. The results showed that the extract, particularly at a dose of 400 mg/kg, significantly reduced paw edema by 26%, supporting the traditional use of *U. dioica* in managing inflammatory conditions (Safari et al. 2016).

**TABLE 4:** Anti‐inflammatory effects of *U. dioica* extracts and diclofenac on carrageenan‐induced paw edema in rats (*n* = 5). Values represent paw diameter measurements over time. Statistically significant differences (*p* < 0.05) were determined in comparison to the negative control and the reference drug (diclofenac, 15 mg/kg).

Treatments	Paw diameter (cm) before carrageenan acid injection	Paw diameter (cm) after carrageenan injection (mean ± SEM)/Percentage inhibition of edema			
		T3	T4	T5	T6
Control	2.02 ± 0.083	2.34 ± 0.09	2.5 ± 0.95	2.44 ± 0.09	2.42 ± 0.04
Diclofenac (15 mg/kg)	2.12 ± 0.13	2.33 ± 0.08	2.31 ± 0.05	2.26 ± 0.05	2.21 ± 0.08
HEE (200 mg/kg)	2.13 ± 0.019	2.37 ± 0.13	2.39 ± 0.022	2.33 ± 0.09	2.30 ± 0.02
HEE (400 mg/kg)	2.12 ± 0.18	2.35 ± 0.22	2.38 ± 0.13	2.32 ± 0.08	2.28 ± 0.07
Poly (50 mg/kg)	2.08 ± 0.12	2.29 ± 0.015	2.31 ± 0.19	2.26 ± 0.010	2.20 ± 0.08
Poly (100 mg/kg)	2.10 ± 0.11	2.30 ± 0.08	2.29 ± 0.14	2.23 ± 0.16	2.21 ± 0.02

Phytochemical screening revealed that *Urtica dioica* extracts are rich in phenolic compounds, particularly chlorogenic acid and caffeic acid, both known for their antioxidant and anti‐inflammatory properties. These compounds may play a role in health promotion and disease prevention, and their synergistic effects warrant further investigation for nutritional and therapeutic applications. In a previous study, caffeic acid demonstrated notable anti‐inflammatory properties. It significantly inhibited nitric oxide (NO) production in LPS‐stimulated RAW 264.7 macrophages, likely through its ability to scavenge NO and suppress inducible nitric oxide synthase (iNOS) expression. These effects were associated with a reduction in inflammatory mediator levels and suggest that caffeic acid can modulate key pathways involved in the inflammatory response (Da Cunha et al. 2004; Elrherabi et al. 2023). Caffeic acid has also demonstrated both antioxidant and anti‐inflammatory properties in a model of noise‐induced hearing loss. It preserved auditory function and reduced cochlear cell death by suppressing oxidative and nitrosative stress and downregulating key inflammatory markers such as NF‐κB and IL‐1β. These findings highlight its potential to protect against cellular damage by modulating redox balance and inflammatory pathways. Caffeic acid is recognized for its cellular protective effects against oxidative stress and inflammation, with evidence supporting neuroprotective benefits that could delay neurodegenerative conditions such as Alzheimer's disease (Kim et al. 2015). Similarly, chlorogenic acid has demonstrated anti‐inflammatory activity and glycemic regulation, making it a compound of interest in both the medical and food industries (Nayeem et al. 2022). This acid exhibits significant anti‐inflammatory activity by targeting multiple inflammatory pathways. It suppresses the production of nitric oxide (NO) and downregulates the expression of key inflammatory enzymes such as, iNOS, and COX‐2. It also reduces the levels of pro‐inflammatory cytokines, including TNF‐α, IL‐1β, and IL‐6 in a dose‐dependent manner. Furthermore, chlorogenic acid inhibits macrophage adhesion, lowers Ninjurin1 (Ninj1) expression, and blocks the nuclear translocation of NF‐κB, highlighting its potential as a therapeutic agent for inflammatory diseases (Hwang et al. 2014). Chlorogenic acid also demonstrates notable anti‐inflammatory and antinociceptive effects in vivo. It significantly reduced carrageenan‐induced paw edema and decreased pain‐related behaviors in the late phase of the formalin test in rats, suggesting an inhibition of peripheral inflammatory mediators. However, CGA did not affect LPS‐induced fever, indicating limited antipyretic activity. These findings support CGA's potential in managing inflammation and pain, though further studies are needed to fully elucidate its mechanisms of action (Dos Santos et al. 2006). These results suggest that polyphenols contribute meaningfully to pain relief, likely through modulation of inflammatory mediators involved in peripheral nociception (Garcia et al. 2015). The dual‐phase formalin test highlighted the capacity of *U. dioica* extracts to reduce both neurogenic and inflammatory pain responses. These effects are likely linked to modulation of key mediators like prostaglandins, serotonin, and histamine (Haley et al. 1989). Anti‐inflammatory assessment using carrageenan‐induced paw edema demonstrated marked inhibition by *U. dioica* extracts, especially at higher doses. Polyphenols like sinapic acid and rutin likely mediate these effects through COX pathway modulation and cytokine inhibition. Additionally, suppression of NF‐κB activation may explain the anti‐inflammatory efficacy observed (Amir and Shikha 2004). In a previous study the hydroalcoholic extract of *Urtica dioica* leaves exhibits significant anti‐inflammatory activity in vivo. In the carrageenan‐induced paw edema model, this extract at a dose of 400 mg/kg reduced paw swelling by 26%, confirming its traditional use for treating inflammatory conditions and highlighting its potential as a natural anti‐inflammatory agent (Hajhashemi and Klooshani 2013).

### 3.4 Acute Safety

An acute toxicity assessment was conducted on the hydroethanolic leaf extract of *Urtica dioica* . The results revealed no signs of toxicity or mortality in any of the treated mice at administered oral doses of 600, 1000, 2000, and 4000 mg/kg. Furthermore, no significant behavioral changes were observed during the 14‐day post‐treatment observation period. Acute toxicity testing revealed no mortality or behavioral changes up to 4000 mg/kg, supporting the safety profile of *U. dioica* extracts. These findings align with previous research indicating non‐toxic properties of this plant (Dar et al. 2013). These findings suggest that the extract exhibits a high safety margin, with no acute toxic effects at doses well above the recommended threshold. According to the OECD Guidelines 423, the absence of clinical symptoms and mortality at such high doses classifies the extract as practically non‐toxic.

### 3.5 Molecular Docking Results

**FIGURE 5:**
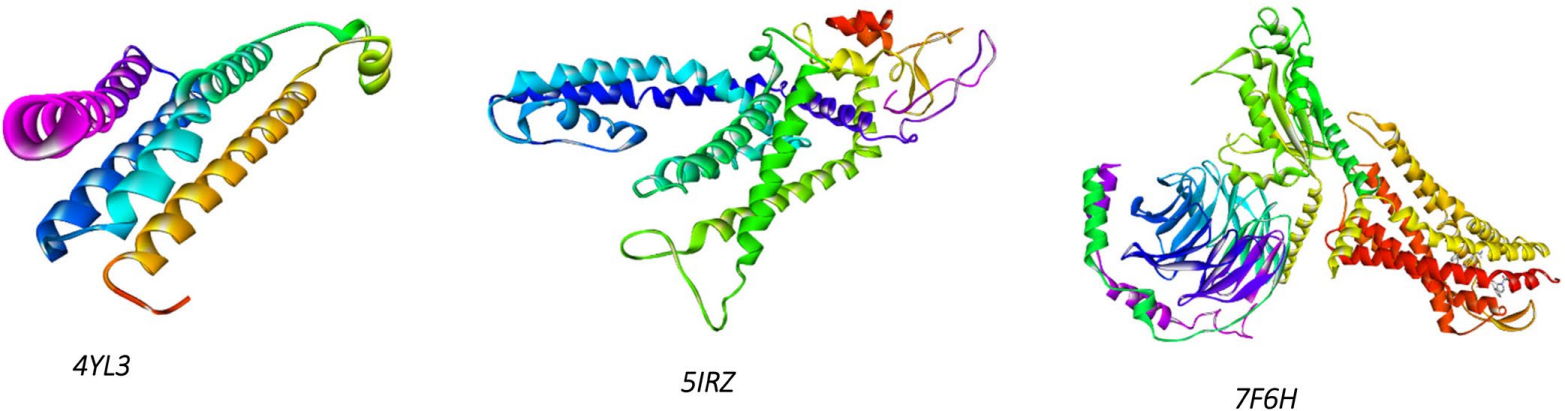
Three‐dimensional ribbon models of the pain‐related targets mPGES‐1 (PDB 4YL3), TRPV1 (PDB 5IRZ) and BK2R (PDB 7F6H).

**FIGURE 6:**
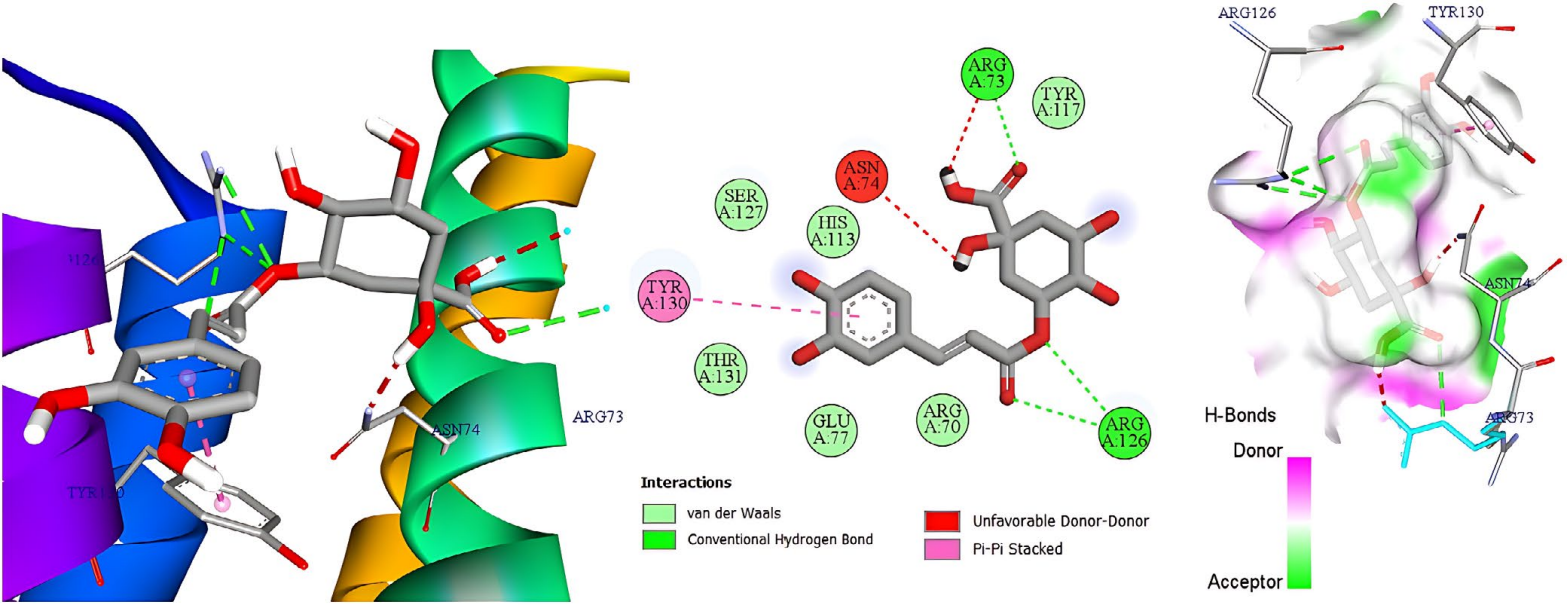
Two‐ and three‐dimensional interaction map including hydrogen‐bond surface of chlorogenic acid bound to mPGES‐1 (PDB 4YL3).

**FIGURE 7:**
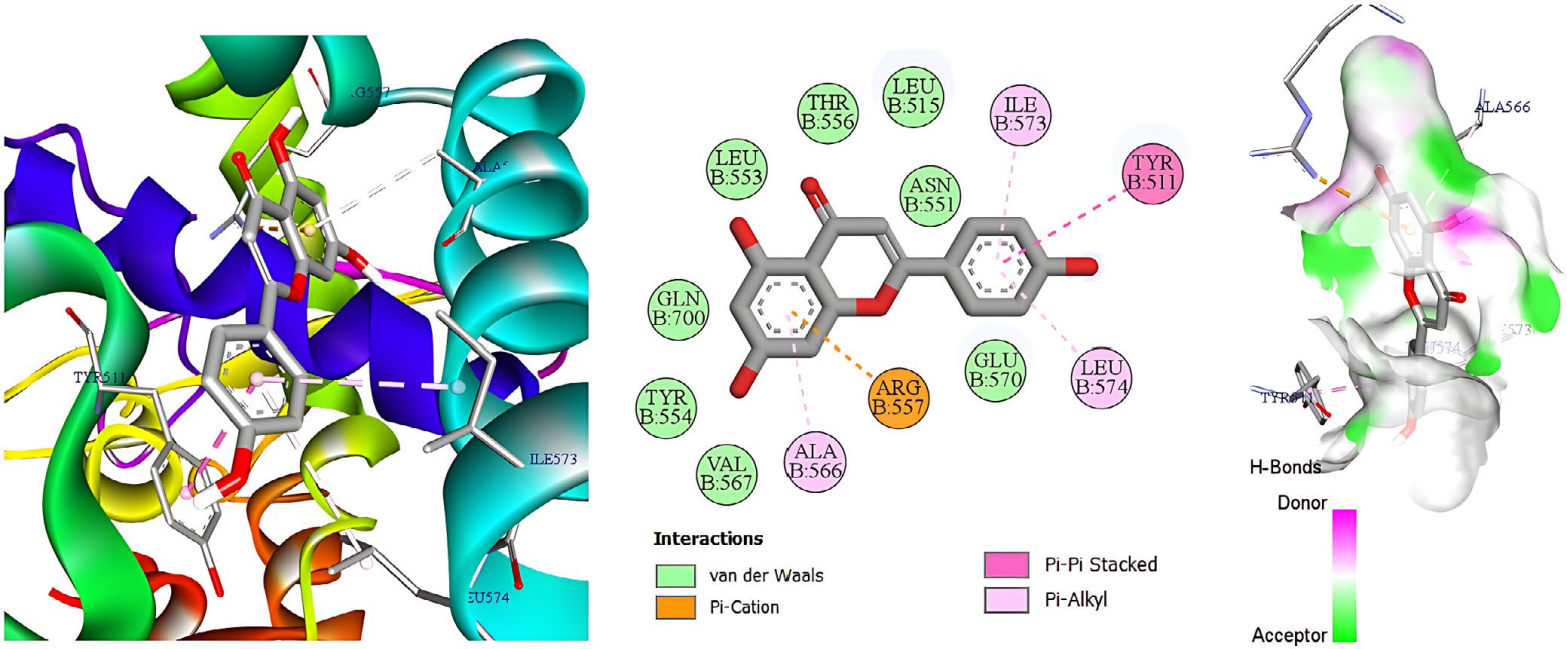
Two‐ and three‐dimensional interaction map including hydrogen‐bond surface of apigenin bound to TRPV1 (PDB 5IRZ).

**FIGURE 8:**
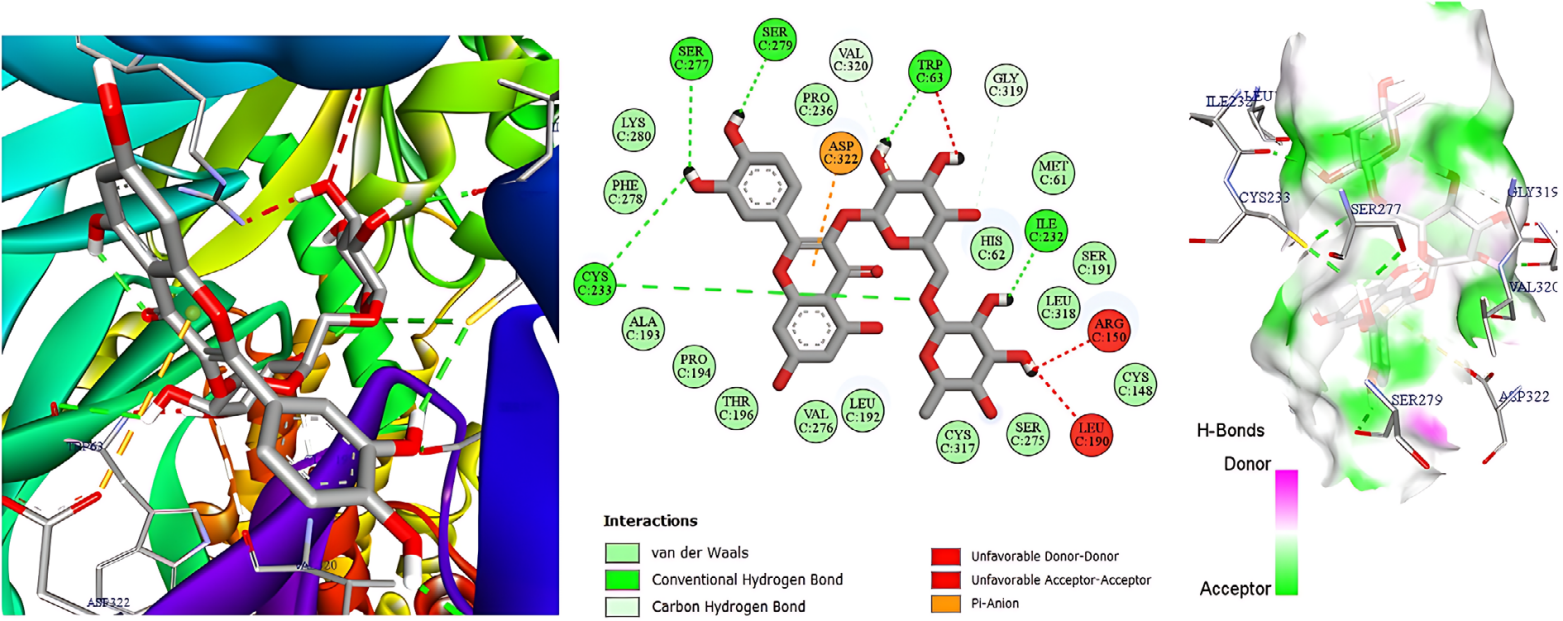
Two‐ and three‐dimensional interaction map including hydrogen‐bond surface of rutin bound to the bradykinin B_2_ receptor BK2R (PDB 7F6H).

Molecular docking was deployed as a physicochemical microscope that aligns the binding landscapes of the nettle‐derived phenolics with those of the clinically validated standards paracetamol (analgesic) and diclofenac (anti‐inflammatory) (Mallet et al. 2023), thereby translating macroscopic bioassays into atom‐scale interaction maps. Guided by our in vivo data, we selected three high‐resolution protein targets that collectively mirror the entire nociceptive cascade, from neurogenic ignition to inflammatory amplification (Figure 5). The acetic‐acid writhing and carrageenan paw‐edema models revealed a dominant dependence on the prostaglandin E2 axis, so microsomal prostaglandin E synthase‐1 (mPGES‐1, PDB 4YL3, 1.41 Å) was chosen to represent this enzymatic choke‐point, with diclofenac providing a pharmacodynamic yardstick (Luz et al. 2015). The immediate antinociception recorded during the early phase of the formalin test pointed to TRPV1 activation, prompting inclusion of the heat‐ and capsaicin‐gated channel (PDB 5IRZ) and comparison with paracetamol as the reference ligand (Gao et al. 2016). The late inflammatory phase of the same assay is driven largely by bradykinin signaling, hence the bradykinin B_2_ receptor (BK2R, PDB 7F6H) was incorporated to complete the triad, again benchmarked against paracetamol to maintain clinical relevance (Shen et al. 2022). This integrated panel furnishes a mechanistically coherent docking framework that not only reconciles our laboratory observations with *in silico* predictions but also isolates the phytochemicals most likely to underpin the extract's dual analgesic and anti‐inflammatory profile. Table 5 indicates that the AutoDock‐Vina scores (kcal mol^−1^) track the extract's in vivo performance with close fidelity. For mPGES‐1 the flavonol glycosides hyperoside, rutin and isoquercitrin, together with the abundant hydroxycinnamate chlorogenic acid (≈−6.6 kcal mol^−1^), bind more favorably than diclofenac (−6.0 kcal mol^−1^). This accords with the 58%–60% inhibition of carrageenan oedema produced by the hydro‐ethanolic extract and the peak 72.5% inhibition achieved by the polyphenolic fraction. On TRPV1, chlorogenic acid tops the list (−8.3 kcal mol^−1^), followed by fisetin (−7.9 kcal mol^−1^) and the flavones luteolin and patuletin (−7.7 kcal mol^−1^ each); all score 2–3 kcal mol^−1^ better than paracetamol (−5.8 kcal mol^−1^), mirroring the ~52% reduction in acetic‐acid writhing elicited by the polyphenolic extract (paracetamol ≈71%). At BK2R, rutin (−10.5 kcal mol^−1^), hyperoside (−9.1 kcal mol^−1^), quercitrin (−9.0 kcal mol^−1^) and luteolin (−8.8 kcal mol^−1^) exceed paracetamol (−5.5 kcal mol^−1^) by ≥ 3 kcal mol^−1^, consistent with the ~60% attenuation of phase‐II formalin pain where bradykinin signaling predominates. Thus, flavonoid scaffolds especially rutin, hyperoside, quercitrin and luteolin emerge as the principal molecular drivers of the nettle extract's analgesic and anti‐inflammatory effects, while chlorogenic and other phenolic acids, by both abundance and respectable affinities, provide complementary, potentially synergistic support. Figures 6, 7, 8 focus on chlorogenic acid, apigenin (−7.6 kcal mol^−1^ on TRPV1) and rutin because this trio best combines quantitative prominence in the extract, scaffold diversity (phenolic acid vs. flavone vs. glycosylated flavonol) and top docking performance on the three pain‐relevant targets (mPGES‐1, TRPV1, BK2R). Visualizing their interactions first will therefore provide the clearest structural basis for follow‐up 2D/3D studies aimed at clarifying how the most abundant and most potent constituents drive the nettle extract's analgesic and anti‐inflammatory effects. Figure 2 illustrates how chlorogenic acid lodges in the mPGES‐1 active site through a triad of classic hydrogen bonds: two symmetric contacts with the guanidinium nitrogens of Arg126 and a third with Arg73, clamping the ester tail deep inside the polar pocket. The three‐dimensional hydrogen‐bond surface in the same figure confirms the fit: a continuous green band (acceptor) hugs the two carbonyl oxygens, while a magenta donor patch at the phenolic OH points toward Arg73; only a small donor‐donor overlap opposite Asn74 marks a minor penalty. Directional stabilization is reinforced by π–π stacking between the caffeoyl ring and Tyr130, and six van‐der‐Waals contacts furnish a snug hydrophobic shell (Et‐Tazy et al. 2025). This blend of triple H‐bonding and aromatic stacking, tempered by a single steric/electrostatic clash, rationalizes the medium docking score (≈−6.6 kcal mol^−1^) and the compound's experimentally moderate yet significant inhibition of mPGES‐1. Figure 3 shows apigenin fitted in the capsaicin pocket of TRPV1 (PDB 5IRZ) through a composite network of non‐covalent forces. Eight van‐der‐Waals contacts provide a snug hydrophobic cradle, while three π‐alkyl interactions with the hydrophobic side chains of Leu574, Ile573 and Ala566 add lateral anchoring. The aromatic system engages in a π‐cation contact with Arg557 and a parallel π–π stack with Tyr511, conferring directional stability inside the channel (Merzouki et al. 2025). The hydrogen‐bond surface (magenta = donor, green = acceptor) displays good polar complementarity: donor and acceptor regions of the ligand neatly overlay reciprocal sites in the pocket, with no notable donor‐donor or acceptor‐acceptor clashes. This blend of aromatic contacts, cation‐π interaction and a uniform van‐der‐Waals envelope rationalizes apigenin's high docking score and supports its proposed role in TRPV1 inhibition and the rapid neurogenic analgesia observed experimentally.

**TABLE 5:** Docking scores (kcal mol^−1^, Vina) for major *Urtica dioica* phenolics and reference drugs against mPGES‐1, TRPV1, and BK2R targets.

Molecules	Docking score (Kcal/mol)		
	4YL3	5IRZ	7F6H
Sinapic acid	−5.2	−6.6	−5.5
Apigenin	−6.4	−7.6	−6.9
Quercitrin	−6.4	−7.6	−9.0
Isoquercitrin	−6.6	−7.3	−7.5
Rutin	−6.6	−7.5	−10.5
Hyperoside	−6.7	−7.4	−9.1
Caffeic acid	−5.3	−6.5	−6.4
Ferulic acid	−5.2	−6.6	−5.7
p‐Coumaric acid	−5.3	−6.2	−5.6
Patuletin	−6.1	−7.7	−8.5
Kaempferol	−6.1	−7.3	−7.0
Luteolin	−6.4	−7.7	−8.8
Chlorogenic acid	−6.6	−8.3	−7.5
Fisetin	−6.3	−7.9	−7.7
Gentisic acid	−5.0	−6.0	−5.7
Diclofenac	−6.0	—	—
Paracetamol	—	−5.8	−5.5

Rutin attains the most favorable affinity in the BK2R series, a result that reflects an unusually dense non‐covalent network within the orthosteric pocket of the receptor. Fifteen van‐der‐Waals contacts mold a hydrophobic cradle around the disaccharide‐flavonol scaffold, while a π‐anion interaction between the conjugated B‐ring and the carboxylate of Asp C322 provides an electrostatic anchor. Two additional C–H···O hydrogen bonds emanate from the rhamnose unit toward Val C320 and Gly C319, tightening the sugar arm against the pocket wall. Figure 4 shows that this core is further stitched in place by six conventional hydrogen bonds: a bidentate motif with the thiol and backbone of Cys C322, single contacts with Ser C277 and Ser C279, and one H‐bond apiece to Trp C63 and Ile C232 on the distal rim. The hydrogen‐bond surface (magenta = donor, green = acceptor) reveals broad complementarity around the ligand; the only mismatches are a donor‐donor clash with Leu C190 and an acceptor‐acceptor overlap with Arg C150, whose energetic penalties are outweighed by the surrounding favorable interactions. Collectively, the π‐anion anchor, the six strong H‐bonds and the hydrophobic shell rationalize the exceptionally low docking score of rutin and support its prominent role in attenuating bradykinin‐mediated pain in vivo.

### 3.6 In Silico ADME Profiling of Chlorogenic Acid, Apigenin and Rutin

**FIGURE 9:**
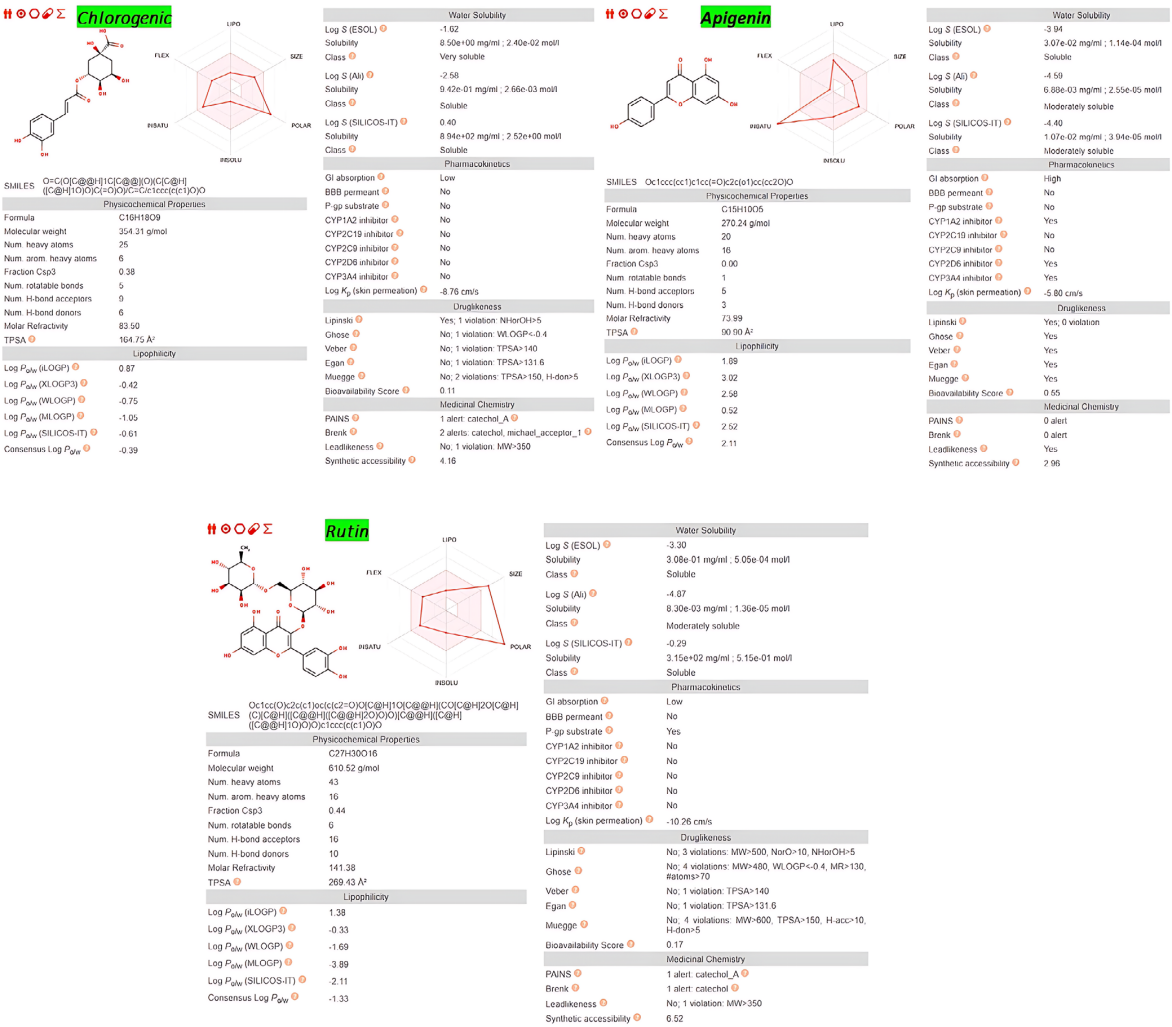
SwissADME radar plots and key numerical descriptors summarizing lipophilicity, polarity, solubility and rule‐based “drug‐likeness” for chlorogenic acid, apigenin and rutin.

Chlorogenic acid, apigenin and rutin exhibit markedly different SwissADME fingerprints that rationalize their pharmacokinetic prospects; Figure 9 encapsulates these data. Chlorogenic acid is highly soluble yet strongly polar (TPSA ≈165 Å^2^; 6 H‐bond donors, 9 acceptors), which explains the “low” oral‐absorption flag, but it incurs only a single Lipinski alert and shows no major CYP‐450 liabilities, implying that suitable formulation could restore systemic exposure. Apigenin offers the most balanced profile: TPSA ≈91 Å^2^ and a consensus cLogP ≈2.1 comply with all Lipinski and Veber rules, yielding a “high” GI‐absorption prediction and the best bioavailability score (0.55), making it the front‐runner for oral development (Lipinski et al. 1997). In contrast, rutin violates four Lipinski criteria because of its large mass (~611 Da) and very high polarity (TPSA ≈269 Å^2^; 10 donors, 16 acceptors); SwissADME therefore forecasts poor absorption and active efflux via P‐gp, indicating that deglycosylation or nano‐carrier strategies would be needed to translate its strong receptor binding into therapeutic exposure.

### 3.7 In Silico Acute Toxicity Assessment Using ProTox‐II

**FIGURE 10:**
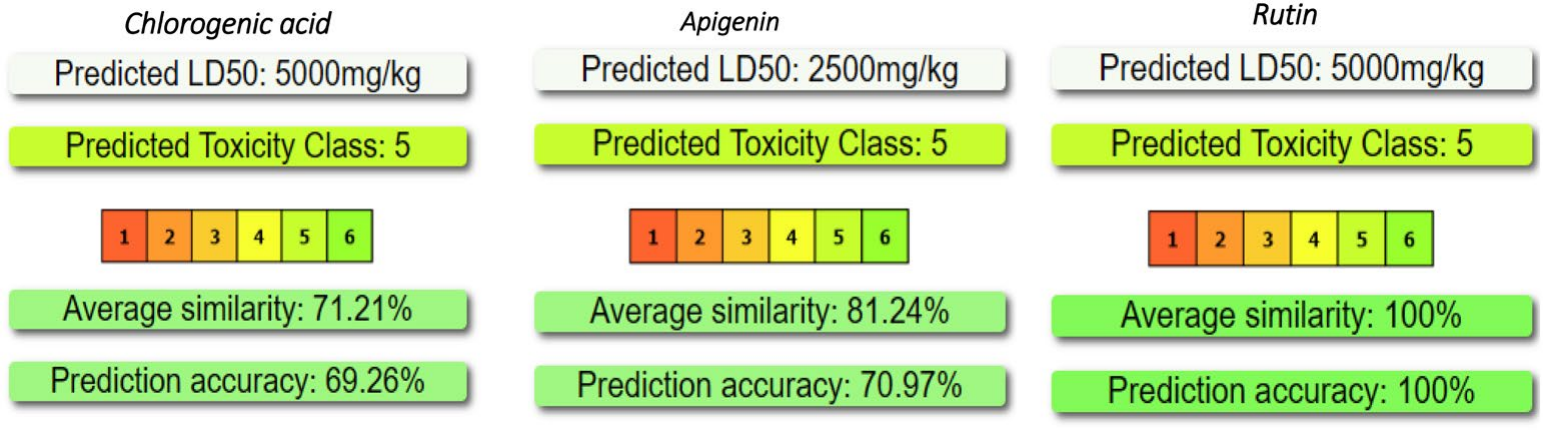
ProTox‐II acute‐toxicity predictions LD_50_ values, OECD toxicity class, and confidence metrics for chlorogenic acid, apigenin, and rutin.

Early‐stage in silico toxicology is essential for narrowing a hit list before animal studies: acute‐toxicity classifiers such as ProTox‐III flag compounds with an unfavorable safety margin, reducing late‐stage attrition and unnecessary in vivo testing. Figure 10 summarizes the ProTox‐III output for chlorogenic acid, apigenin and rutin. All three fall into toxicity class 5 (LD_50_ = 2000–5000 mg kg^−1^), indicating low acute oral toxicity by OECD standards (Suwardi et al. 2023). Chlorogenic acid and rutin yield a predicted LD_50_ of 5000 mg kg^−1^, placing them at the benign end of class 5, whereas apigenin is somewhat more active toxically with an LD_50_ of 2500 mg kg^−1^. The similarity scores (71% for chlorogenic acid, 81% for apigenin, 100% for rutin) reflect how closely each molecule matches non‐toxic reference structures in the training set; the associated prediction accuracies (≈69%–100%) reinforce confidence that none approaches the more hazardous classes 1–4. Taken together, the data suggest that the three leads possess a wide acute‐safety window, supporting their progression to sub‐chronic and mechanistic toxicity assays that will be required for definitive risk assessment.

The chosen protein panel mPGES‐1, the TRPV1 ion channel, and the BK2R receptor logically aligns with the biological pathways highlighted by our in vivo assays, allowing us to trace the extract's antinociceptive and anti‐inflammatory effects down to the atomic level. Glycosylated flavonoids (especially rutin, hyperoside, and isoquercetin) bind to mPGES‐1 with lower free energies than the reference drug diclofenac, whereas chlorogenic acid shows the strongest affinity for TRPV1. Rutin again ranks first against BK2R, reaching a binding energy of −10.5 kcal mol^−1^. This hierarchy mirrors the carrageenan‐induced oedema and formalin test inhibition recorded in vivo and pinpoints rutin, hyperoside, and luteolin as the main molecular drivers of nettle activity, while phenolic acids contribute abundance and synergistic support to the overall efficacy.

SwissADME fingerprints reveal striking differences in drug‐likeness among the key compounds. Chlorogenic acid is highly water‐soluble, yet its pronounced polarity limits oral uptake. Apigenin, by contrast, combines moderate polarity (TPSA ≈91 Å^2^) and a balanced cLogP (~2.1) with no Lipinski warnings, giving it the best predicted bioavailability and oral developability. Rutin violates four Lipinski rules because of its large mass and high polarity, suggesting that advanced delivery strategies will be needed to ensure systemic exposure. ProTox‐III classifies all three molecules in acute‐toxicity class V (LD_50_ = 2000–5000 mg kg^−1^), with chlorogenic acid and rutin offering the widest safety margins and supporting their progression to sub‐chronic and mechanistic toxicity studies. Hydro‐ethanolic maceration recovers the full spectrum of *Urtica dioica* phenolics and flavonoids dominated by caffeic and chlorogenic acids plus rutin, apigenin and quercetin that synergistically quench free radicals and suppress pain‐ and inflammation‐related pathways. Bioassays show the polyphenol‐enriched fraction provides stronger peripheral and central analgesia and greater oedema reduction than the crude extract, while molecular docking reveals a multitarget mode of action centred on mPGES‐1, TRPV1 and BK2R, with glycosylated flavonoids and phenolic acids occupying the top‐affinity poses. ADME fingerprints predict good oral absorption for most constituents (rutin excepted, requiring tailored delivery), and acute‐toxicity studies report no mortality or behavioral changes, indicating a wide safety margin. Together, these data establish nettle as a safe, multitarget phytopharmaceutical source that couples antioxidant, anti‐inflammatory and analgesic efficacy, meriting further development into standardized nutraceuticals and therapeutic formulations (Carvalho et al. 2017).

## 4 Conclusions

The present study was carried out to evaluate and valorize *U. dioica* , an aromatic and medicinal plant, widely used in traditional medicine in Morocco. We have tried to highlight the main chemical compounds and biological activities. Findings showed that *U. dioica* had a variety of secondary metabolites. Both of methanolic extract and the polyphenolic fraction of *Urtica dioica* provide therapeutic evidence in anti‐inflammatory and analgesic effects. The results of this study justified the traditional use of the plant; it is an interesting source of biologically active compounds that may be applied for therapy in humans. Additionally, the richness of data derived from this study may be useful as a solid basis for further research on *Urtica dioica* , by valorizing its potential utility in various fields, including medical and cosmetic sciences. Further, deeper and more complete research will be needed to clarify the chemical properties and mechanisms of action of the active principles extracted and to exploit the studied biological properties in the pharmaceutical field in order to make products based on natural molecules.

## Author Contributions

Conceptualization, S.T., and A.L., and O.K.; methodology, S.T., A.L., and O.K.; software, O.K. and S.T.; validation, A.E., and M.B.; formal analysis, S.T., and A.L.; investigation, S.T., M.A., and A.A.Q.; resources, A.L., and A.A.Q; data curation, S.T., M.D., and O.K.; writing – original draft preparation, O.K., S.T., A.E., M.B., and A.L.; writing review and editing, A.E., M.B., O.K., M.A., F.A.N., A.A.Q., A.L., and M.D.; visualization, A.E., and M.B.; supervision, H.B.; funding acquisition, F.A.N.; all authors have read and agreed to the published version of the manuscript.

## Funding

This work was supported and funded by the Deanship of Scientific Research at Imam Mohammad Ibn Saud Islamic University (IMSIU) (grant number IMSIU‐DDRSP2501).

## Disclosure

The authors hereby declare that the work presented in this article is original and that any liability for claims relating to the content of this article will be borne by them.

## Ethics Statement

All animal experiments were conducted in accordance with internationally recognized ethical standards, including the European Directive 86/609/EEC and the National Institutes of Health (NIH) Guide for the Care and Use of Laboratory Animals (NIH Publication No. 85‐23, revised 1985). The study protocol was reviewed and approved by the Institutional Ethics Committee for the Care and Use of Laboratory Animals at the Faculty of Medicine and Pharmacy of Fez, Sidi Mohamed Ben Abdellah University, under ethical approval number 05/2023/LERHS. All necessary measures were taken to minimize animal suffering and to ensure compliance with the principles of humane care and use of laboratory animals.

## Conflicts of Interest

The authors declare no conflicts of interest.

## Data Availability

The original contributions presented in the study are included in the article. Further inquiries can be directed to the corresponding author.
